# Handedness and Laterality in Plastic Surgery and Outcome—A Retrospective, Two-Center, Evaluator-Blinded Study

**DOI:** 10.1007/s00266-025-04868-y

**Published:** 2025-04-28

**Authors:** Flurina Gass, Alice Thürlimann, Mauro Maniglio, Carlo M. Oranges, Daniel Kalbermatten, Mathias Tremp

**Affiliations:** 1 Faculty of Medicine, University of Basel, Basel, BS Switzerland; 2 Department of Plastic, Reconstructive and Aesthetic Surgery, Geneva University Hospitals (HUG), University of Geneva, Geneva, Switzerland; 3 Private Practice, Hirslanden Private Hospital Group, Dorfplatz 1, 6330 Cham, Switzerland

**Keywords:** Functional laterality, Patient outcome assessment, Comparative study, Complications, Retrospective studies

## Abstract

**Background:**

Little is known about the influence of handedness on the outcome of operations in plastic surgery. Our study addresses the question of whether there is a difference between the right and left sides in the outcome of plastic surgeries in relation to the handedness of the surgeon.

**Patients and Methods:**

In this retrospective two-center study, patients undergoing plastic surgery on bilateral locations (breast reduction, mastopexy, augmentation mastopexy, blepharoplasty, mastectomy on gynecomastia, brachioplasty, thigh lift) between January 2020 and December 2023 were included. The outcome was assessed separately by an independent assessor. The Likert scale (1–10) was used as the standardized assessment method for the study data. Additionally, complications were classified as minor or major complications, depending on whether a reoperation was necessary or not.

**Results:**

During the study period, 61 patients (four men and 57 women) were included (mean age 44 years, range 18—83 years) with a mean follow-up of 9.3 months (range 1—28 months). There was no statistically significant difference between both sides (*p *= 0.60) with a positive trend toward the right side. There were no major complications and 12 minor complications (19.7%) with a tendency to a higher complication rate on the right side.

**Conclusion:**

Our results indicate that handedness may play a minor role in the outcome of plastic surgeries. Nevertheless, ergonomic measurements can be made in order to optimize the outcome. Moreover, it might be necessary to include ambiguity in handedness to improve the overall outcome.

**Level of Evidence IV:**

This journal requires that authors assign a level of evidence to each article. For a full description of these Evidence-Based Medicine ratings, please refer to the Table of Contents or the online Instructions to Authors  www.springer.com/00266.

## Introduction

Small asymmetries regarding the aesthetic outcome in bilateral operation sites are common and part of the informed preoperative consent. The assumption of the surgical outcome of the operative site corresponding to the dominant hand (e.g., right breast operated by a right-handed surgeon) being usually slightly better is a subjective statement we share with other authors [1]. It is commonly known that plastic surgeons prefer operating on one side of the body rather than the other side due to the position of the dominant hand, making surgery easier and more economic [1]. A survey showed a preference for the right breast in 60% stated by right-handed plastic surgeons [1]. Perhaps the reason for the predilection of operating on the side ipsilateral the surgeon’s dominant hand relates to the ergonomically easier access and maneuvering of instruments on the ipsilateral side [2].

In the current literature, there are no scientific data available supporting the hypothesis that outcomes are better on the side corresponding to the surgeon’s handedness for procedures in plastic surgery. In contrast, operating on the ipsilateral side of the surgeon’s dominant hand resulting in better surgical outcomes has been observed in other surgical specialties [2–7]. Studies have shown that arthroplasties resulted in better outcome with less complications for the right hip or knee if a surgeon is right-handed and inversely [3–6, 8]. The same evidence exists for Otorhinolaryngology (ENT) for right-handed surgeons operating on the right ear having better outcomes in terms of operative duration and hearing improvement compared to right-handed surgeons operating on the left ear and reciprocally [2].

Reasons for the different outcomes between the two sites depending on handedness have not been determined but may be related to dexterity or proprioception being better when working with the dominant hand [5].

That being said, little is known in the literature in plastic surgery about the influence on the postoperative outcome depending on surgeon’s handedness and laterality of the operative site. Ultimately, those factors are clinically important, because they can help gauge success rates and estimate duration of operations. They may also be relevant to the patient because shorter operative times would also mean shorter anesthesia time and consequently less concomitant complications. Thus, the aim of this retrospective study was to analyze the influence of the surgeon’s handedness on the aesthetic outcome and complication rate in patients undergoing plastic procedures on two parallel sites of the body.

## Patients and Methods

Patients undergoing plastic surgery on bilateral locations from January 2020 to December 2023 were included. The outcome was assessed separately on each side by an independent and trained assessor. All surgeries were performed by board-certified right-handed plastic surgeons with more than 10 years of experience, and for each side the surgeon was standing on the corresponding surgical field. The following surgeries were included: breast reduction, mastopexy, augmentation mastopexy, blepharoplasty, mastectomy on gynecomastia, brachioplasty and thigh lift. Outcome measures were the Likert scale/visual analog scale (VAS) [9]. It has been shown that the Likert scale of measuring satisfaction has a good validity and reliability and can be used in clinical trials due to the ease of administration and interpretation [9]. Complications were assessed as minor or major complications, depending on whether a reoperation was necessary or not, and according to the Clavien–Dindo classification [10–12]. Eventually, the results were summarized, pooled together and averaged. Written informed consent was obtained from all patients, and the guidelines of the Declaration of Helsinki were followed accordingly. Ethical approval was obtained by the local ethical committee (Ethikkommission Nordwest- und Zentralschweiz (EKNZ), No.: 2024-00364).

### Statistical Analysis

Data are presented as frequencies for categorical variables, means and standard deviation (SD) for normally distributed variables and median and range for not normally distributed variables where appropriate. The normality of data distribution was verified using the Kolmogorov–Smirnov test. Descriptive statistics described the population characteristics. The Mann–Whitney test (nonparametric) was used to compare the scores between subgroups that were considered as independent samples. Statistical significance was determined by a value of *p* ≤ 0.05. Analyses were performed using GraphPad Prism version 5.00 for Windows (GraphPad Software, San Diego, CA, USA).

## Results

### Likert Scores

During the study period, 61 patients (four men and 57 women) were included (mean age 44 years, range 18–83 years) with a mean follow-up of 9.3 months (range 1–28 months). Totally there were 40 patients with breast surgeries (17 breast reductions, seven breast augmentations, six mastopexies, four breast implant exchanges, three augmentations with mastopexy and three mastectomies on gynecomastia), 19 patients with upper blepharoplasties, one thigh lift and one brachioplasty, respectively. All procedures had been performed bilaterally.

There was the biggest absolute difference in Likert scores although statistically not significant in between the two sides of 0.75 for breast implant exchange with capsulectomy in favor of the right side (Likert score of 9.5 for the right and 8.75 for the left side). For most breast surgeries, such as breast augmentation with or without mastopexy as well as thigh lifts and brachioplasties, no difference in Likert scores for the two sites was noted. Interestingly, for breast reductions and mastopexies, Likert scores for the left side were minimally higher (Table 1).

**Table 1:** Summary of surgeries and associated procedures

	Breast augmentation	Capsulectomy and breast implant exchange	Breast augmentation mastopexy	Breast reduction	Mastopexy	Mastectomy on Gynecomastia	Blepharoplasty	Thigh lift	Brachioplasty
Patients (N)	7	4	3	17	6	3	19	1	1
Likert scale, mean (SD^a^), IQR^b^	R: 9.43 (0.53), 1 L: 9.43 (0.79), 1	R: 9.5 (0.58), 1 L: 8.75 (0.5), 0.5	R: 8.67 (0.58), 1 L: 8.67 (0.58), 1	R: 8.65 (1), 1 L: 8.7 (1.05), 1	R: 9.17 (0.75), 1 L: 9.33 (0.52), 1	R: 10 (0), 0 L: 10 (0), 0	R: 9.42 (0.84), 1 L: 9.37 (0.68), 1	R: 10, 0 L: 10, 0	R: 10, 0 L: 10, 0
Age, mean (SD), IQR	37.14 (9.1), 16	59.25 (10.37), 14.5	23.33 (1.15), 2	39.12 (16.31), 29	39.5 (4.28), 6	31.67 (22.81), 40	56.47 (11.2), 8	35, 0	53, 0

^a^*SD* standard deviation, ^b^*IQR* interquartile range

**Fig. 1:**
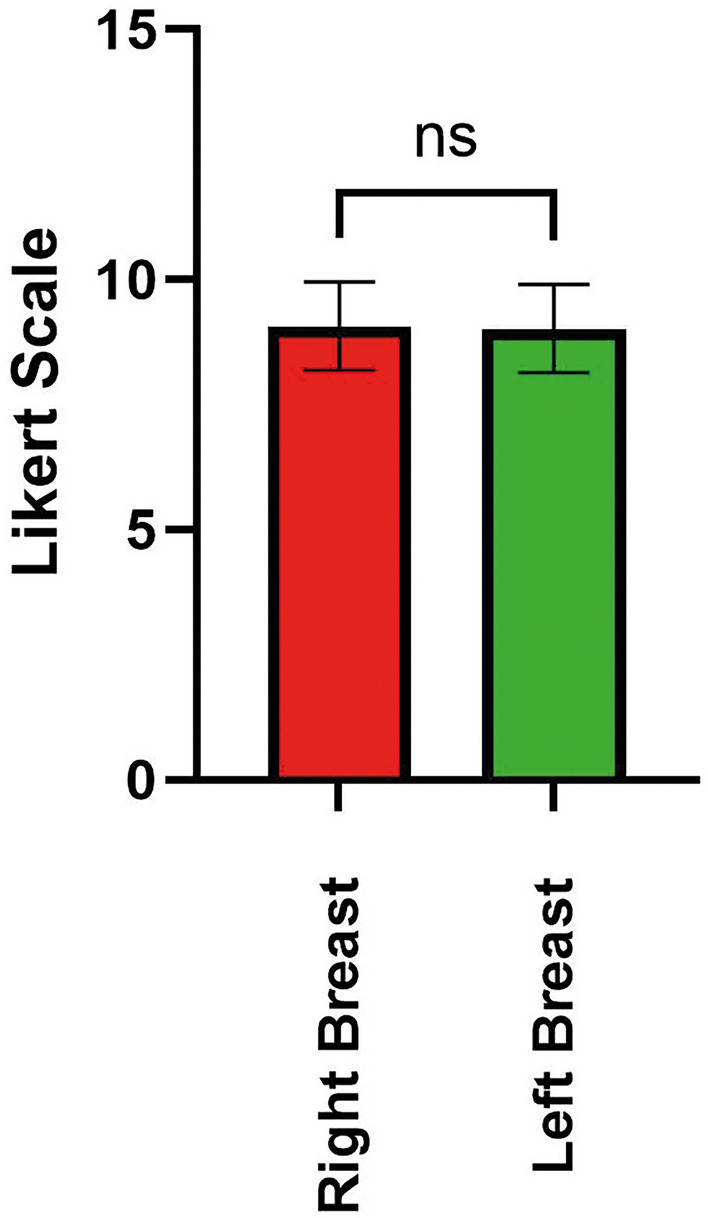
Bar plot with Likert scores for the breast surgery group

**Fig. 2:**
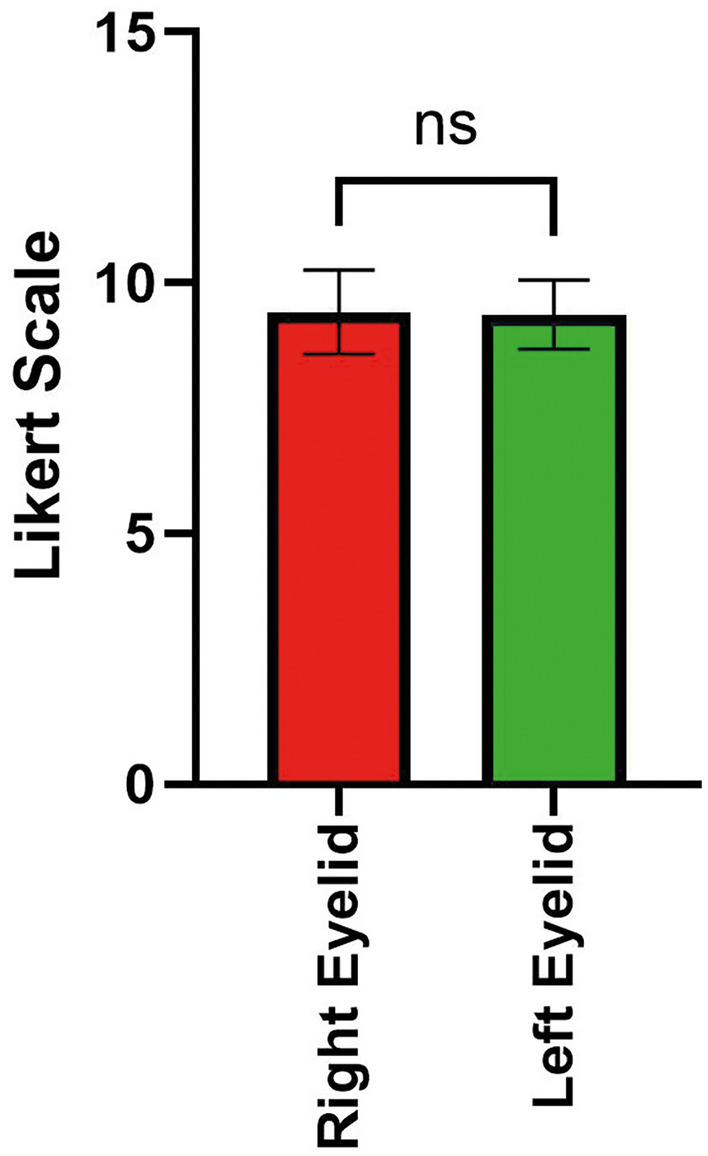
Bar plot with Likert scores for the blepharoplasty surgery group

There was no statistically significant difference in the overall outcome between both sides (*p* = 0.60), although with a positive trend toward the right side. Specifically, there was no statistically significant difference in the outcome among the overall breast surgeries (Likert scale 9.07 ± 0.88 right side, Likert scale 9.02 ± 0.88 left side, *p* = 0.80, Fig. 1). A smaller almost negligible difference in results in favor to the right side was attained for upper blepharoplasties (9.42 ± 0.84 right side, 9.37 ± 0.68 left side, *p* = 0.68, Fig. 2).

### Complications

The results on the Clavien–Dindo scale (I–V) showed a mean value of 1.29. There were no major complications and 12 minor complications (19.7%) with a tendency to a higher complication rate on the right side: wound healing delay and wound dehiscence in five patients on the right side and in four patients on the left side, one infected lymphocele in the right axilla after excision of ectopic breast tissue, one hematoma in the right breast after breast reduction and one abscess after breast reduction.

### Representative Patients

#### Patient 1

**Fig. 3:**
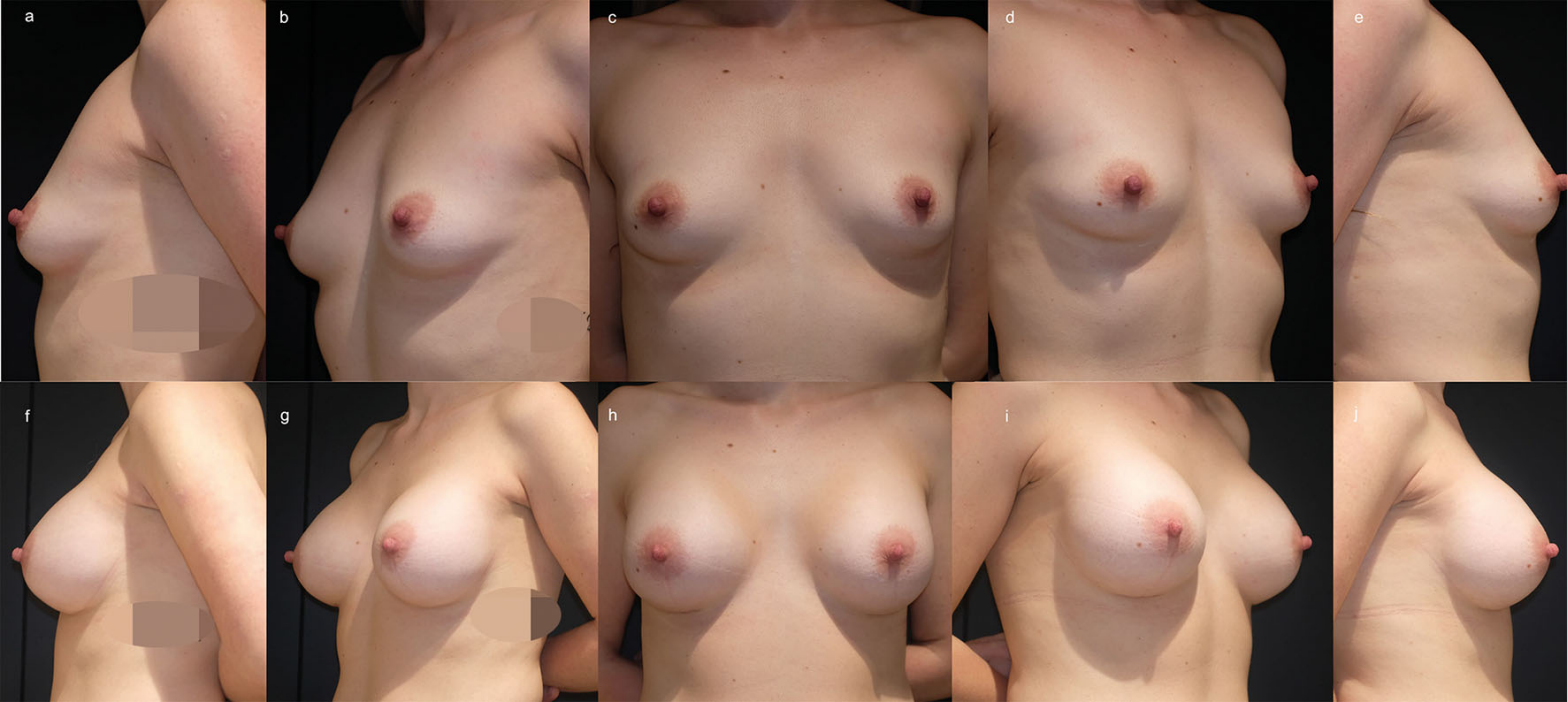
A 33-year-old woman with breast hypoplasia and involution (**a**–**e**) after pregnancy underwent bilateral submuscular breast augmentation dual plane II (320 cc (Ref.: E2SF-320Q, Motiva)). The postoperative course was uneventful. After a follow- up of six months (**f**–**j**), clinical analysis showed a window shading effect on the left side. Nevertheless, the patient was very satisfied with the overall outcome (VAS 10 on both sides). Our independent evaluation confirmed the patient’s satisfaction (Likert scale 10 on both sides), and the scar was imperceptible

A 33-year-old woman after pregnancy underwent bilateral submuscular dual plane II breast augmentation (320 cc (Ref.: E2SF-320Q, Motiva)). The postoperative course was uneventful. After a follow-up of six months, clinical analysis showed a window shading effect on the left side. Nevertheless, the patient was very satisfied with the overall outcome (VAS 10 on both sides). Our independent evaluation confirmed the patient’s satisfaction (Likert scale 10 on both sides), and the scar was imperceptible (Fig. 3).

#### Patient 2

**Fig. 4:**
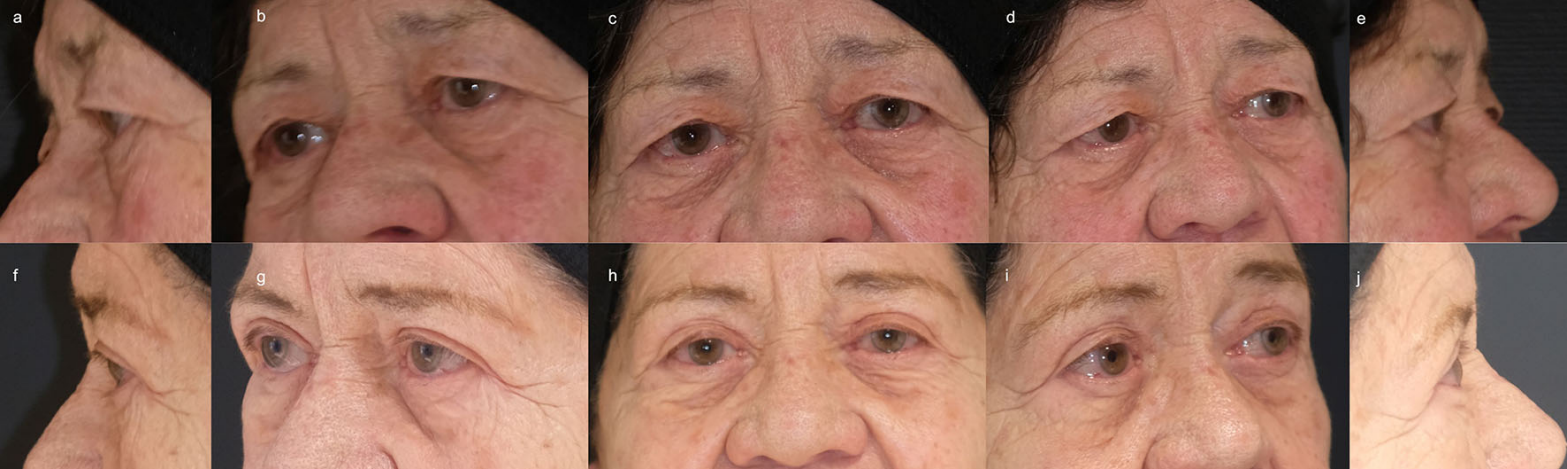
An 83-year-old woman underwent bilateral upper blepharoplasty with severe hooding and skin excess (**a**—**e**). The postoperative course was uneventful and there were no complications. After a follow-up of 6.3 months (**f**—**j**), clinical analysis showed a satisfactory result, which goes in line with patient satisfaction (VAS 10 on both sides). Our independent evaluation confirmed the patient’s satisfaction (Likert scale 10 on both sides), and the scar was imperceptible

An 83-year-old woman underwent bilateral upper blepharoplasty with severe hooding and skin excess. The postoperative course was uneventful and there were no complications. After a follow-up of 6.3 months, clinical analysis showed a satisfactory result, which goes in line with patient satisfaction (VAS 10 on both sides). Our independent evaluation confirmed the patient’s satisfaction (Likert scale 10 on both sides), and the scar was imperceptible (Fig. 4).

#### Patient 3

**Fig. 5:**
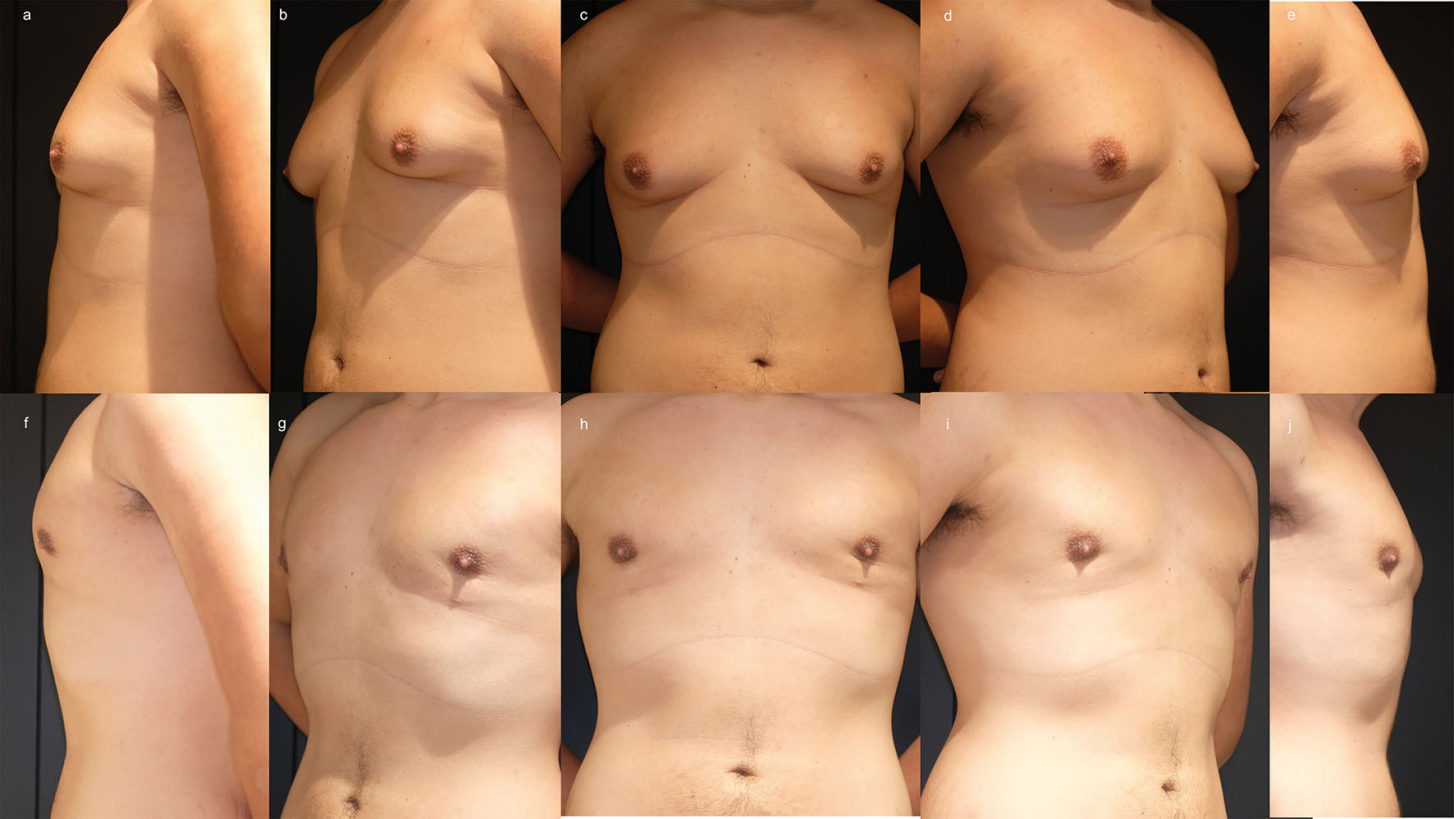
A 19-year-old man underwent bilateral water-assisted liposuction and pull-through technique with drains for the treatment of Grade 3 [22] gynecomastia as previously described (**a**—**e**) [13]. The postoperative course was uneventful and there were no complications. After a follow-up of 12 months, clinical analysis showed a satisfactory result (**f**—**j**) with minimal indentation on the left side which did not bother the patient. Patient satisfaction was VAS 10 for both sides. Our independent evaluation confirmed the patient’s satisfaction (Likert scale 10 on both sides), and the scar was imperceptible

A 19-year-old man underwent bilateral water-assisted liposuction and pull-through technique with drains for the treatment of gynecomastia as previously described [13]. The postoperative course was uneventful and there were no complications. After a follow-up of 12 months, clinical analysis showed a satisfactory result, which goes in line with patient satisfaction (VAS 10 on both sides). Our independent evaluation confirmed the patient’s satisfaction (Likert scale 10 on both sides), and the scar was imperceptible (Fig. 5).

#### Patient 4

A 25-year-old female underwent bilateral breast reduction with a super-medial pedicle, circumvertical incisions and along the inframammary fold.

**Fig. 6:**
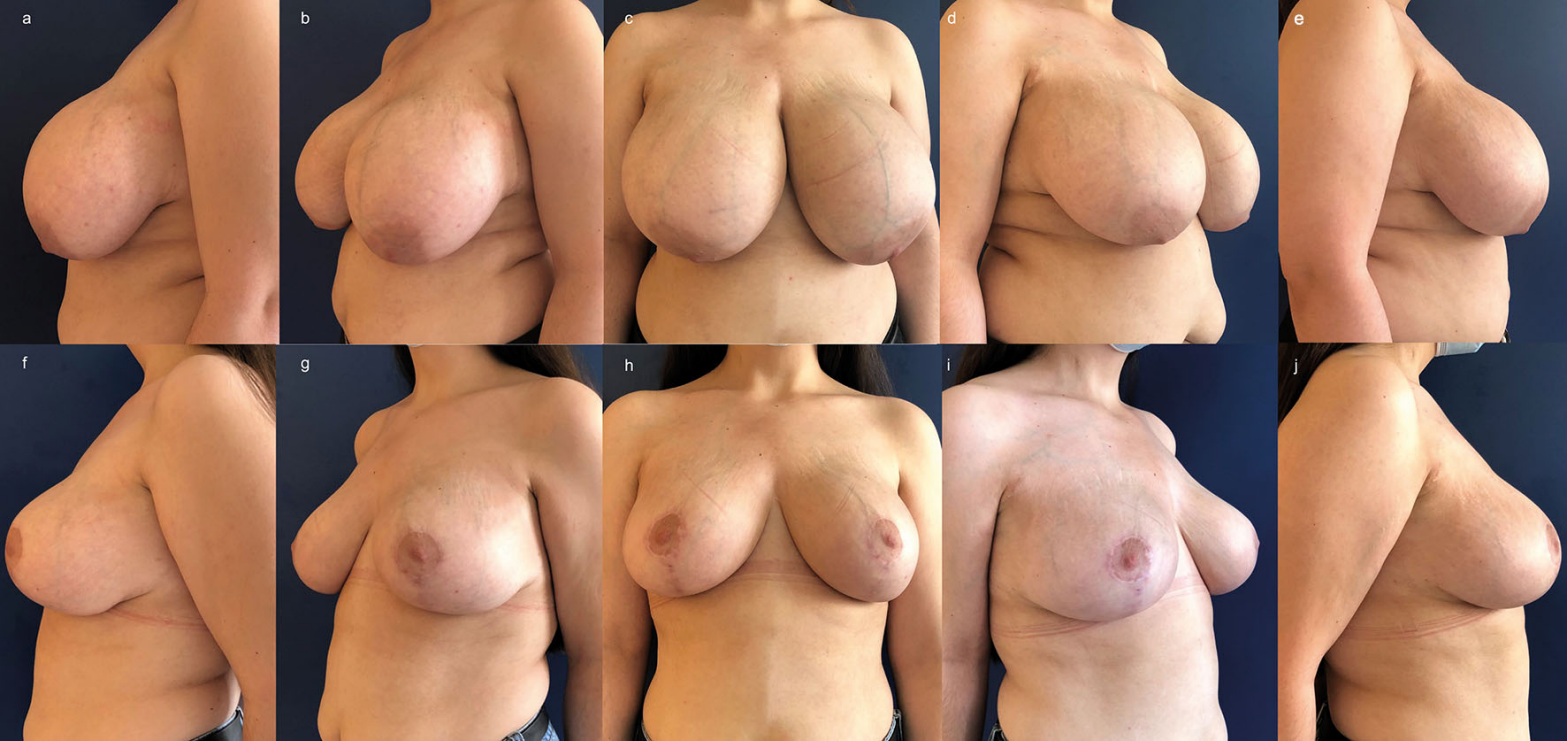
A 25-year-old female with macromastia (**a**—**e**) underwent bilateral breast reduction with a super-medial pedicle, circumvertical incisions and along the inframammary fold. The patient had previous radiotherapy as a child for Hodgkin lymphoma. The postoperative course was uneventful without any complications. The patient was satisfied with the result (**f**—**j**) six months after the surgery but was slightly displeased by the hypopigmented periareolar scar. Our independent evaluation showed a Likert score of 7 for the right side and a Likert score of 9 for the left side

The patient had previous radiotherapy as a child for Hodgkin lymphoma. The postoperative course was uneventful without any complications. The patient was satisfied with the result at 6 month postoperatively but was slightly displeased by the hypopigmented periareolar scar. Our independent evaluation showed a Likert score of 7 for the right side and a Likert score of 9 for the left side (Fig. 6).

## Discussion

In this retrospective study, we were able to break the dogma that the outcome of the right side is similar to the left side in right-handed plastic surgeon. Even though we received minimally higher Likert scores on the right side for patients with capsulectomy and implant exchanges, and almost negligible higher scores for breast augmentation and blepharoplasties, they are all statistically insignificant. For the results in breast reduction and breast mastopexies, we received even minimally better overall Likert scores for the left side.

The comparison of outcomes of aesthetic and plastic procedures to the ones of orthopedics or other surgical fields is nearly impossible since the parameters are vastly different. While we evaluated Likert scales based on the aesthetic appearance of postoperative results, there are functional outcomes, measurements in radiologic images and occurrence of specific complications assessed in orthopedics, neurosurgery or oncologic breast surgery.

Complication rates would be comparable among surgical specialties if classified according to Clavien–Dindo, as we did for our adverse events. Orthopedic papers stated that handedness has an impact of the overall outcome and complication rate such as for unicompartmental knee arthroplasty [5]. Surgeon handedness may cause a worse prosthetic orientation on the femoral side during the surgeon’s nondominant unicompartmental knee arthroplasty [5]. In another study, surgeon handedness appears to influence acetabular component position during total hip replacement but it is one factor of many that interact to achieve a successful outcome [6]. Also, in spine surgery surgeon handedness appears to have an influence over the orientation of pedicle screws [14]. This may create problems for right-handed surgeons in the insertion of upper-level screws from the left side and lower-level screws from the right side [14].

Conversely, Luvisa et al. found a higher rate of complications after mastectomy, including seroma, hematoma, infection and ischemia, in the ipsilateral breast [15]. Examining complication rates, they found the greatest impact on ischemic complications after mastectomy being the surgeon’s years of experience. With less than 15 years of experience they had a 2.7 times increase in any ischemic complications in mastectomy and a 16 times increase of major ischemic complications needing a reoperation [15]. This fact emphasizes the value of experience of a surgeon over their handedness.

The acquirement of ambidexterity is desirable for many surgical disciplines. For left-handed physicians, it is an absolute necessity with most instruments built for usage by right-handed individuals [16–20].

In a survey by Ramsden et al., self-ambidexterity was as high as 5.3% among plastic surgeons, whereas the frequency in the general population is 1% [1]. The prevalence of functional ambidexterity is possibly higher in surgeons due to the nature of their training and practice over the years, which can mitigate the differences between the dominant and nondominant hand.

Nevertheless, being ambidextrous does not guarantee better skills compared to being exclusively right- or left-handed. Yet, it is generally recognized that training the nondominant hand is a crucial component of the curriculum [1].

Ambidexterity can be trained by forcing oneself to use both hands equally and consistently while operating, which moreover comes along with cortical reorganization [21]. Ambidexterity and various established measures, such as patient positioning and ergonomic adaptions, clearly may optimize outcomes on the contralateral side. For instance, a right-handed surgeon might stand on the right side of the patient and adjust the operating table toward their side to work on the left side effectively.

Nonetheless, our study has limitations, such as being retrospective with a small sample size on a single evaluation tool with a relatively short follow-up and a lack of homogeneity. Furthermore, the study did not investigate how the outcome varies with a left-handed surgeon, volume (surgeries done per year) and experience of the surgeon. Further studies are needed with a larger sample size, potentially a future randomized controlled trial with a long-term follow-up on one type of surgical procedure only, and more specific quality of life (QoL) and patient-reported outcome measures (PROM) tools (e.g., BREAST-Q scores) as well as operative time per side to further validate our findings.

## Conclusion

To the best of our knowledge, this is the first study to examine how surgeon handedness and surgical site laterality may impact the overall outcome in plastic surgery. Our preliminary results indicate that handedness may play a minor role in the overall outcome of plastic surgeries and that the results are similar regardless of the handedness of the operating surgeon.

## References

[1] Ramsden AJ. Plastic surgeons: are we dextrous or sinister? J Plast Reconstr Aesthet Surg. 2012;65:402–3. 10.1016/j.bjps.2011.08.024. 21889917 10.1016/j.bjps.2011.08.024

[2] Barzaga G. The relationship of surgeon handedness and experience on operative duration and hearing improvement in ipsilateral and contralateral otologic surgeries. Philippine J Otolaryngol Head Neck Surg. 2020;35:17.

[3] Kong X, Yang M, Li X, et al. Impact of surgeon handedness in manual and robot-assisted total hip arthroplasty. J Orthop Surg Res. 2020;15:159. 10.1186/s13018-020-01671-0. 32316973 10.1186/s13018-020-01671-0 PMC7171772

[4] Kong X, Yang M, Ong A, et al. A surgeon’s handedness in direct anterior approach-hip replacement. BMC Musculoskelet Disord. 2020;21:516. 10.1186/s12891-020-03545-2. 32746833 10.1186/s12891-020-03545-2 PMC7397678

[5] Mehta S, Lotke PA. Impact of surgeon handedness and laterality on outcomes of total knee arthroplasties: should right-handed surgeons do only right TKAs? Am J Orthop (Belle Mead NJ). 2007;36:530–3. 18033564

[6] Pennington N, Redmond A, Stewart T, Stone M. The impact of surgeon handedness in total hip replacement. Ann R Coll Surg Engl. 2014;96:437–41. 10.1308/003588414X13946184902488. 25198975 10.1308/003588414X13946184902488 PMC4474195

[7] Yaman O, Acaroğlu E. Role of surgeon handedness in transpedicular screw insertion. Acta Orthop Traumatol Turc. 2014;48:479–82. 10.3944/AOTT.2014.13.0046. 25429570 10.3944/AOTT.2014.13.0046

[8] Cao Z, Liu Y, Yang M, et al. Effects of surgeon handedness on the outcomes of unicompartmental knee arthroplasty: a single center’s experience. Orthop Surg. 2022;14:3293–9. 10.1111/os.13549. 36281639 10.1111/os.13549 PMC9732585

[9] Guyatt GH, Townsend M, Berman LB, Keller JL. A comparison of Likert and visual analogue scales for measuring change in function. J Chronic Dis. 1987;40:1129–33. 10.1016/0021-9681(87)90080-4. 3680471 10.1016/0021-9681(87)90080-4

[10] Dindo D, Demartines N, Clavien P-A. Classification of surgical complications. Ann Surg. 2004;240:205–13. 10.1097/01.sla.0000133083.54934.ae. 15273542 10.1097/01.sla.0000133083.54934.ae PMC1360123

[11] Clavien PA, Barkun J, de Oliveira ML, et al. The Clavien–Dindo classification of surgical complications: five-year experience. Ann Surg. 2009;250:187–96. 10.1097/SLA.0b013e3181b13ca2. 19638912 10.1097/SLA.0b013e3181b13ca2

[12] Katayama H, Kurokawa Y, Nakamura K, et al. Extended Clavien-Dindo classification of surgical complications: Japan clinical oncology group postoperative complications criteria. Surg Today. 2016;46:668–85. 10.1007/s00595-015-1236-x. 26289837 10.1007/s00595-015-1236-x PMC4848327

[13] Lista F, Ahmad J. Power-assisted liposuction and the pull-through technique for the treatment of gynecomastia. Plast Reconstr Surg. 2008;121:740–7. 10.1097/01.prs.0000299907.04502.2f. 18317124 10.1097/01.prs.0000299907.04502.2f

[14] Yaman O. Role of surgeon handedness in transpedicular screw insertion. Acta Orthop Traumatol Turc. 2014;48:479–82. 10.3944/AOTT.2014.13.0046. 25429570 10.3944/AOTT.2014.13.0046

[15] Luvisa K, Fan KL, Black CK, et al. Does surgeon handedness or experience predict immediate complications after mastectomy? A critical examination of outcomes in a single health system. Breast J. 2020;26:376–83. 10.1111/tbj.13487. 31448506 10.1111/tbj.13487

[16] Anderson M, Carballo E, Hughes D, et al. Challenges training left-handed surgeons. Am J Surg. 2017;214:554–7. 10.1016/j.amjsurg.2016.12.011. 28108068 10.1016/j.amjsurg.2016.12.011

[17] Denison ME, Awad K, Gillen JR, et al. Issues and strategies in training left-handed surgeons. Am Surg. 2023;89:5107–11. 10.1177/00031348231175119. 37212798 10.1177/00031348231175119

[18] Rohrich RJ. Left-handedness in plastic surgery: asset or liability? Plast Reconstr Surg. 2021;148:8S-9S. 10.1097/01.prs.0000794764.90878.fb. 34699469 10.1097/01.prs.0000794764.90878.fb

[19] Savetsky IL, Cammarata MJ, Kantar RS, et al. The left-handed plastic surgery trainee: perspectives and recommendations. Plast Reconstr Surg Glob Open. 2020;8:e2686. 10.1097/GOX.0000000000002686. 33133882 10.1097/GOX.0000000000002686 PMC7572112

[20] Shay T, Kaufman T, Cohen AA, Ad-El D. Is being left handed an advantage toward a plastic surgery residency? Plast Reconstr Surg Glob Open. 2020;8:e2589. 10.1097/GOX.0000000000002589. 32095399 10.1097/GOX.0000000000002589 PMC7015585

[21] Ramesh SV, Ramesh PV, Ray P, et al. Training ambidexterity—A survey-based analysis on the dexterity of ophthalmologists in performing standard ophthalmic procedures. Indian J Ophthalmol. 2023;71:2947–52. 10.4103/IJO.IJO_3315_22. 37530262 10.4103/IJO.IJO_3315_22 PMC10538830

[22] Simon BE, Hoffman S, Kahn S. Classification and surgical correction of gynecomastia. Plast Reconstr Surg. 1973;51:48–52. 10.1097/00006534-197301000-00009. 4687568 10.1097/00006534-197301000-00009

